# Isolation and *in vitro* characterization of bacterial isolates from fermented pearl millet

**DOI:** 10.3389/fmicb.2026.1835809

**Published:** 2026-06-25

**Authors:** Janani Latha Ravi, Sandeep Singh Rana

**Affiliations:** Department of Bioscience, School of Biosciences and Technology, Vellore Institute of Technology, Vellore, India

**Keywords:** antagonistic activity, antioxidant activity, bacterial isolates, fermented pearl millet, *Lactiplantibacillus plantarum*, *Pediococcus pentosaceus*

## Abstract

**Introduction:**

This study aimed to isolate and characterize bacterial strains from fermented pearl millet and evaluate their *in vitro* characteristics.

**Methods:**

A total of 66 bacterial colonies were isolated, of which three representative isolates (PJ2, PJ8, and PJ23) were selected for further analysis based on the preliminary screening criteria. Based on 16S rRNA gene sequencing, the isolates were identified as *Bacillus aryabhattai*, *Pediococcus pentosaceus*, and *Lactiplantibacillus plantarum*.

**Results and discussion:**

The selected isolates demonstrated tolerance to acidic pH and bile salt conditions, with survival rates ranging from 50.3 to 88.8% at pH 2.0 and 42.7 to 83.5% following exposure to 0.3% bile salts for 1 to 3 h of incubation. Under the tested *in vitro* conditions, the isolates exhibited antagonistic activity against foodborne pathogens, antioxidant activity, phenotypic exopolysaccharide-associated characteristics, and varying levels of cell surface hydrophobicity. *Pediococcus pentosaceus* PJ8 exhibited the highest DPPH radical scavenging activity (62.3%). In agar well diffusion assays, *Lactiplantibacillus plantarum* PJ23 demonstrated inhibitory activity against *Escherichia coli* (29.3 ± 4.0 mm), whereas *Pediococcus pentosaceus* PJ8 exhibited inhibitory activity against *Bacillus cereus* (35 ± 5 mm) and *Staphylococcus aureus* (40 ± 5 mm). Among the isolates, *Lactiplantibacillus plantarum* PJ23 displayed comparatively higher cell surface hydrophobicity values (45.7–50.3%). Overall, the findings demonstrated that the selected bacterial isolates exhibited preliminary *in vitro* characteristics under the conditions tested.

## 1 Introduction

Microorganisms associated with fermented foods have attracted considerable scientific and industrial interest because of their diverse technological and functional characteristics (Reuben et al., 2020; Rahman et al., 2026). Among them, lactic acid bacteria (LAB) are widely associated with fermented foods, dairy, and beverage systems owing to their long history of use and technological relevance (Lashani et al., 2020; Salim and Jumali, 2020; Khusro et al., 2021; Mohanan et al., 2025; Rahman et al., 2026). The growing interest in cereal- and millet-based fermented products has increased research on the bacteria associated with these fermentation systems (Ruwala and Shaikh, 2023; Janani and Adeyeye, 2025). Compared with bacteria commonly associated with dairy-based fermented products, cereal- and millet-associated bacteria remain comparatively underexplored, despite the growing interest in plant-based fermented foods (Bembem and Agrahar-Murugkar, 2020; D’Almeida et al., 2024).

Pearl millet (*Pennisetum glaucum*) is the sixth most important cereal crop globally and serves as a staple food for millions of people in Asia and Africa (Satyavathi et al., 2021). It is widely cultivated in arid and semi-arid regions because of its tolerance to drought and low-input agricultural conditions (Yadav et al., 2012; Krishnan and Meera, 2018). Pearl millet contains dietary fiber, proteins, lipids, vitamins, and minerals such as iron, zinc, phosphorus, potassium, and magnesium, and considerable quantities of sulfur-containing amino acids, including methionine and cysteine (Rao et al., 2006; Poornachandra Rao et al., 2015; Uppal et al., 2015). Its gluten-free nature and low glycemic index further support its application in dietary interventions for individuals with gluten intolerance, diabetes, and other metabolic disorders (Satyavathi et al., 2021). The nutrient-rich composition and fermentable carbohydrate profile of pearl millet may also support the growth and metabolic activity of lactic acid bacteria (LAB) during spontaneous fermentation (Satyavathi et al., 2021; Gouthami et al., 2024).

Fermentation of cereals and millets has long been practiced as a traditional strategy to improve their shelf life, sensory quality, and nutritional availability (Arya et al., 2025). During fermentation, microbial metabolism contributes to acidification, degradation of anti-nutritional factors, and formation of microbial metabolites (Arya et al., 2025). Earlier studies have demonstrated that LAB fermentation reduces tannin and phytic acid levels in cereals and millets, thereby improving digestibility and nutrient bioavailability (Chavan et al., 1988; Lorri and Svanberg, 1995). (Gouthami et al., 2024) reported that native LAB isolated from fermented finger millet, Kodo millet, and little millet included members of the genera *Lactobacillus*, *Lactiplantibacillus*, *Pediococcus*, *Weissella*, and *Enterococcus*. The study also demonstrated the antibacterial and antifungal activities of selected *Weissella* isolates, including the inhibition of *Aspergillus flavus*, highlighting the potential role of millet-associated LAB in contributing to microbial stability in fermented cereal-based foods. Representative microorganisms previously isolated from fermented millet and cereal-based products and their characteristics are summarized in Table 1.

**Table 1:** Representative microorganisms reported in fermented millet and cereal-based products and their functional characteristics.

Microorganism	Fermented source	Reported functional characteristics	Reference
*Lactobacillus plantarum*	Fermented millet and cereal products	Acid tolerance, antagonistic activity, and reported in vitro traits	(Poornachandra Rao et al., 2015; Gouthami et al., 2024)
*Lactobacillus fermentum*	Fermented finger millet	Salt tolerance and in vitro functional characteristics	(Gouthami et al., 2024)
*Pediococcus acidilactici*	Fermented millet products	Exopolysaccharide production and reported functional characteristics	(Gouthami et al., 2024)
*Weissella cibaria*	Fermented millet products	Inhibitory effects, bile tolerance, and hydrophobicity	(Manovina et al., 2022; Gouthami et al., 2024)
*Weissella confusa*	Fermented millet products	Antifungal and antagonistic activity	(Gouthami et al., 2024; Nath et al., 2025)
*Lacticaseibacillus rhamnosus*	Fermented cereal batters	In vitro characterization	(Divyashree et al., 2024)
*Lacticaseibacillus casei*	Fermented cereal batters	Acid tolerance and antagonistic activity	(Divyashree et al., 2024)

Acid and bile tolerance are commonly evaluated during the characterization of bacterial isolates associated with fermented foods and probiotic-related research, as survival under acidic and bile stress conditions is considered important in simulated gastrointestinal environments (Naeem et al., 2024). Similarly, cell surface hydrophobicity has been suggested as a preliminary indicator of microbial surface interaction properties under *in vitro* conditions (Gouthami et al., 2024). In addition to survival characteristics, increasing attention has been directed toward the metabolites and antagonistic compounds produced by LAB during fermentation. LAB interact with food substrates through glycolysis, lipolysis, and proteolysis, resulting in the release of organic acids, peptides, amino acids, fatty acids, and bacteriocins (Kahieshesfandiari et al., 2021; Vasiee et al., 2022); (Divyashree et al., 2024) isolated *Lactiplantibacillus plantarum*, *Lacticaseibacillus paracasei*, and *Limosilactobacillus fermentum* from traditional multigrain millet dosa batter and reported several functional characteristics under *in vitro* conditions, including survivability under simulated gastric conditions, cholesterol assimilation, and inhibitory activity of LAB-derived cell-free supernatants against foodborne pathogens. Furthermore, (Dopazo et al., 2024) demonstrated that LAB fermentation of cereal-derived agro-industrial substrates resulted in the production of antifungal metabolites, including lactic acid and phenyllactic acid, which contributed to the inhibition of food spoilage fungi and the reduction of aflatoxin occurrence.

Therefore, the present study aimed to isolate and characterize bacterial isolates obtained from fermented pearl millet under in vitro conditions. The isolates were evaluated for acid and bile tolerance, antioxidant activity, antagonistic activity, cell surface hydrophobicity, and antibiotic susceptibility.

## 2 Materials and methods

### 2.1 Sample collection and preparation

Local landrace pearl millet (collected from the Vellore region, Tamil Nadu, India) and hybrid pearl millet (Rocket 9,990 variety) were procured from a local supermarket in Vellore, Tamil Nadu, India. The seeds were manually cleaned to remove dust, debris, and any damaged seeds. The cleaned seeds were stored in airtight containers at room temperature until further analysis. The cleaned seeds were used for physical and proximate characterization and for preparing a fermented pearl millet slurry for the isolation of potential bacterial isolates.

### 2.2 Physical and proximate characterization of pearl millet

Before fermentation, local and hybrid pearl millet samples were evaluated for their physical and proximate characteristics.

#### 2.2.1 Determination of physical characteristics

##### 2.2.1.1 Seed weight

In this method, 1,000 grains of each native and hybrid variety were randomly counted and weighed individually in triplicate on an electronic scale with a minimal sensitivity of 0.01 g, and the findings were recorded in grams (Kaur, 2021).

##### 2.2.1.2 Seed density

Fifty grams of seeds were precisely weighed and placed in a measuring cylinder. It was then filled with 50 mL of distilled water. The seed volume was determined by subtracting 50 mL from the total volume. Finally, the density was determined using the formula (Nelson, 2002).

##### 2.2.1.3 Seed volume

Fifty seeds from each millet type were placed in a 50 mL measuring container, followed by 25 mL of distilled water. The difference in total volume (25/50) was measured three times (Mohsenin, 1970).

##### 2.2.1.4 Bulk density

A predefined quantity of the sample was placed in a previously weighed 5 mL measuring cylinder (W1), lightly tapped to avoid air gaps between the flour that was in the measuring cylinder, and the volume was recorded (W2). The new mass of the sample was calculated (Mohsenin, 1970). The bulk density was calculated as:


$$\text{Bulk density}=\frac{W2-W1}{\text{Volume of seed}}$$

##### 2.2.1.5 Hydration capacity

Seeds weighing 50 g were counted and placed in a measuring cylinder. 150 mL of water was added to the cylinder, which was then covered with the foil and left at room temperature overnight. The next day, the seeds were drained, excess water was filtered out using filter paper, and the swollen seeds were reweighed (Nithyashree and Vijayalaxmi, 2023).

##### 2.2.1.6 Hydration index

The formula for calculating the hydration index was as follows (Nithyashree and Vijayalaxmi, 2023):


$$\text{Hydration index}=\frac{\text{Hydration capacity}\;\mathrm{per}\;\text{seed}}{\text{Weight}\;(g)\;\text{of}\;\mathrm{one}\;\text{seed}}$$

##### 2.2.1.7 Swelling capacity

Seeds weighing 50 g were counted, their volume was measured, and they were soaked overnight. The volume of the soaked seeds was measured using a graduated cylinder:


$$\text{Swelling capacity}=\frac{\text{Volume after soaking}-\text{Volume before soaking}}{50}$$

##### 2.2.1.8 Swelling index

The formula for calculating the swelling index was as follows: (Nithyashree and Vijayalaxmi, 2023):


$$\text{Swelling index}=\frac{\text{Swelling capacity}\;\mathrm{per}\;\text{seed}}{\text{Volume}\;(\mathrm{mL})\;\text{of}\;\mathrm{one}\;\text{seed}}$$

##### 2.2.1.9 Germination percentage

To evaluate germination percentage, randomly picked seeds were soaked overnight and placed on wet filter paper on Petri plates. All samples were incubated at 37 °C for at least 48 h. In the meantime, the paper was kept wet with a sprinkle of water, and the germination percentage was measured for each type of millet (Yenasew and Urga, 2023):


$$\text{Germination percentage}=\frac{\text{Seeds germinated}}{\text{Total seeds}}\times 100$$

#### 2.2.2 Proximate composition analysis

##### 2.2.2.1 Moisture content

The moisture content was determined according to (Association of Official Analytical Chemists, 2000).

Briefly, 5 g of the sample was dried in a hot air oven at 105 °C for 6 h. After cooling in a desiccator, the moisture content was calculated using the following equation:


$$\text{Moisture}\;(\%)=\frac{\text{Loss in weight}\;(g)}{\text{Weight of sample}\;(g)}\times 100$$

##### 2.2.2.2 Crude protein

Crude protein content was determined using the Kjeldahl method according to (Association of Official Analytical Chemists, 2000). Briefly, the samples were digested with concentrated sulfuric acid in the presence of a catalyst to convert organic nitrogen into ammonium sulfate. The digested sample was then neutralized and distilled, and the liberated ammonia was collected and titrated with a standard acid solution. The crude protein content was calculated using the following equation:


$$\text{Crude protein}\;(\%)=\frac{0.00014\times V\times (S-B)\times 100}{V1\times W}\times F$$

Where, *W* = weight of the sample (g); *V* = total volume of digest (mL); V1 = volume of digest taken for distillation (mL); S = volume (mL) of standard acid used for sample titration; *B* = volume (mL) of standard acid used for blank titration; 0.00014 = nitrogen equivalent factor; *F* = conversion factor (6.25) used to convert nitrogen to crude protein.

##### 2.2.2.3 Crude fat

Crude fat content was determined using a Soxhlet extraction apparatus with petroleum ether as the extraction solvent, according to (Association of Official Analytical Chemists, 2000). The extracted fat was quantified gravimetrically, and the crude fat content was calculated using the following equation:


$$\mathrm{Fat}\;(\%)=\frac{\text{Loss of weight}\;(g)}{\text{Sample weight}\;(g)}\times 100$$

##### 2.2.2.4 Crude fiber

The crude fiber content was determined by the acid and alkali digestion method following the procedure described by (Feldsine et al., 2002). Briefly, the sample was sequentially digested with 1.25% sulfuric acid, followed by 1.25% sodium hydroxide. The digested residue was filtered, dried, and incinerated in a crucible. The difference in weight before and after ashing of the digested residue was used to calculate the crude fiber content using the following equation:


$$\text{Crude fiber}(\%)=\frac{W2-W3}{W1}\times 100$$

W1 represents the weight of the sample (in grams); W2 = weight of insoluble matter (weight of crucible + insoluble matter − weight of crucible); W3 = ash weight (crucible weight + ash weight − crucible weight).

##### 2.2.2.5 Ash content

A five-gram sample was weighed into a crucible and heated over a modest Bunsen burner flame with the lid half open. As soon as the fumes subsided, the crucible and lid were placed in a furnace and heated at 550 °C overnight without covering the lid. After complete combustion, a lid was applied to prevent the loss of the fluffy ash and left to cool. When the sample turned grey, the ash was weighed using a crucible and lid. The percentage ash content was calculated using the procedure (Association of Official Analytical Chemists, 2000).


$$\mathrm{Ash}\;(\%)=\frac{\text{Loss of weight}\;(g)}{\text{Sample weight}\;(g)}\times 100$$

### 2.3 Isolation and preliminary characterization of bacterial isolates

Pearl millet slurry was prepared by mixing pearl millet flour with distilled water at a 1:4 (w/v) ratio (25 g flour in 100 mL water). The slurry was allowed to ferment spontaneously for 48 h under anaerobic conditions at room temperature. The pH was measured at 0, 24, and 48 h using a digital pH meter (Anil, 2021). Following fermentation, the samples were serially diluted tenfold using sterile distilled water, and the appropriate dilutions were spread plated onto de Man, Rogosa, and Sharpe (MRS) agar (HiMedia Laboratories Pvt. Ltd., Mumbai, India). The medium was prepared according to the manufacturer’s instructions and sterilized before plating. The plates were then incubated anaerobically at 37 °C for 48 h. Colonies exhibiting distinct morphological characteristics were selected and repeatedly sub-cultured on fresh MRS agar plates using the streak plate method to obtain pure cultures. The purified isolates were maintained on MRS agar slants at 4 °C for further analysis (Amanda et al., 2025). The isolates were preliminarily screened based on colony morphology, Gram staining, catalase activity, and microscopic characteristics. Gram staining was performed to identify Gram-positive bacteria, a catalase test was performed to confirm catalase-negative isolates, and cell morphology was observed microscopically using a light microscope (Khushboo et al., 2023).

### 2.4 Biochemical characterization of isolates

Standard biochemical tests, including methyl red (MR), Voges–Proskauer (VP), hydrogen sulfide production, indole production, glucose fermentation with gas production, ammonia production, nitrate reduction, and citrate utilization tests, were performed for the isolates using standard microbiological procedures and media (HiMedia Laboratories Pvt. Ltd., Mumbai, India). The inoculated media were incubated at 37 °C for 24–48 h, depending on the test requirements. The obtained biochemical characteristics were interpreted and compared with the descriptions provided in Bergey’s Manual of Determinative Bacteriology (Buchanan and Gibbons, 1974). Isolates that were Gram-positive, catalase-negative, and had related phenotypic and biochemical characteristics were selected for further functional evaluation.

### 2.5 Molecular identification of selected isolates

Genomic DNA was obtained from the selected isolates using the EXpure Microbial DNA Isolation Kit (Bogar Bio Bee Stores Pvt. Ltd., India). Polymerase Chain Reaction (PCR) was used to amplify the 16S rRNA gene using universal primers 27F (5′-AGAGTTTGATCCTGGCTCAG-3′) and 1492R (5′-TACGGTACCTTGTTACGACTT-3′). PCR amplification was performed under standard amplification conditions with initial denaturation at 95 °C for 2 min, followed by 25 cycles of denaturation at 95 °C for 30 s, annealing at 50 °C for 30 s, extension at 72 °C for 2 min, and final extension at 72 °C for 10 min. The product was confirmed through 1% agarose gel electrophoresis and visualized under UV illumination. The purified product was subjected to bidirectional sequencing using an ABI 3730xl Genetic Analyzer (Applied Biosystems, United States). Sequencing was performed by Yaazh Xenomics (Coimbatore, India) and Eurofins Genomics India Pvt. Ltd. The obtained sequences were compared using the NCBI BLAST program. Phylogenetic analysis was performed to confirm the taxonomic identification of the isolates. The obtained sequences were submitted to GenBank and assigned accession numbers.

### 2.6 Functional characterization of isolates

#### 2.6.1 Acid tolerance (pH 2 and 4 survival)

The isolates were grown overnight in MRS broth (HiMedia Laboratories Pvt. Ltd., India) under anaerobic conditions at 37 °C. The cultures were centrifuged at 7000 rpm for 10 min and washed twice with sterile phosphate-buffered saline (PBS) before analysis. Sterile MRS broth was adjusted to pH 2.0 and 4.0 using 1 M HCl. Briefly, 100 μL of the washed cell suspension was inoculated into 900 μL of acidified MRS broth, and the initial viable counts (0 h) were recorded before incubation. The cultures were incubated at 37 °C with agitation for 3 h. Samples were collected at 1 h intervals, serially diluted with sterile phosphate-buffered saline (PBS) or normal saline, and viable counts were determined using the pour plate method. The survival rate (%) was calculated by comparing the viable counts at each time point to the initial count (0 h) (Gore, 2017; Chen et al., 2023; Biswas et al., 2026).

#### 2.6.2 Bile salt tolerance (0.3% bile)

The isolates were grown overnight in 10 mL of MRS broth (HiMedia Laboratories Pvt. Ltd., India) under anaerobic conditions at 37 °C. Approximately 0.1 mL of culture suspension was inoculated into 0.9 mL of MRS broth supplemented with 0.3% (w/v) bile salts (Sigma-Aldrich, United States; Product No. 48305-50G-F), while the MRS broth without bile salts served as the control. The cultures were incubated at 37 °C for 3 h. Samples were collected at 1 h intervals, serially diluted with sterile normal saline, and plated onto MRS agar using the pour-plate technique. After incubation at 37 °C for 24–48 h, viable cells were counted. The initial viable counts (0 h) were recorded before incubation. Survival rate (%) was calculated by comparing viable counts at each time point to the initial count (0 h) (Gore, 2017; Chen et al., 2023).

#### 2.6.3 Exopolysaccharide (EPS) production

The strains were streaked onto MRS agar plates (HiMedia Laboratories Pvt. Ltd., India) supplemented with 5% glucose and incubated at 37 °C for 48 h to screen for exopolysaccharide (EPS)-associated phenotypic characteristics. Mucoid colony formation and slime production were evaluated using the loop test method as preliminary phenotypic indicators commonly associated with exopolysaccharide formation (Patel et al., 2012).

#### 2.6.4 Antioxidant activity (DPPH radical scavenging assay)

The antioxidant activity of the isolated cultures was evaluated using the DPPH (2,2-diphenyl-1-picrylhydrazyl) radical scavenging assay (Kuskoski et al., 2005). Cell-free culture supernatants were obtained by centrifuging overnight cultures at 7000 rpm for 10 min, and the supernatants were collected for analysis. The cell-free supernatants were tested at different concentrations (10, 20, and 30%). Briefly, 50 μL of each sample was mixed with 100 μL of 0.4 mM DPPH solution and incubated in the dark at room temperature for 30 min. The absorbance was measured at 517 nm using a UV–Vis spectrophotometer (UV-1900, Shimadzu, Japan). Ascorbic acid was used as a standard antioxidant for the comparison. The DPPH scavenging activity (%) was calculated using the following equation:


$$\text{DPPH scavenging activity}\;(\%)=(\frac{A_{\text{control}}-A_{\text{sample}}}{A_{\text{control}}})\times 100$$

#### 2.6.5 Cell surface hydrophobicity

Cell surface hydrophobicity of the isolates was evaluated using chloroform, ethyl acetate, and xylene as solvents according to the method described by (Rosenberg et al., 1980). Briefly, 3 mL of bacterial culture was mixed with 1 mL of each solvent and vortexed for 1 min. The mixture was allowed to separate into two phases at room temperature, and the aqueous phase was carefully collected. Absorbance was measured at 600 nm using a UV–Vis spectrophotometer (UV-1900, Shimadzu, Japan). Hydrophobicity was calculated using the following equation:


$$\text{Hydrophobicity}\;(\%)=1-\frac{A_{\text{Final}}}{A_{\text{initial}}}\times 100$$

#### 2.6.6 Antibiotic resistance testing

The antibiotic susceptibility of the isolates was evaluated using the disc diffusion method, as previously described for lactic acid bacteria susceptibility studies (Charteris et al., 1998). Antibiotic discs containing ampicillin, gentamicin, streptomycin, kanamycin, and erythromycin (HiMedia Laboratories Pvt. Ltd., India) were used for the study. The cultures were inoculated onto Mueller–Hinton (MH) agar plates (HiMedia Laboratories Pvt. Ltd., India) and incubated at 37 °C for 24–48 h. The diameter of the inhibition zones was measured using a Vernier caliper.

#### 2.6.7 Agar well diffusion assay against foodborne pathogens

The inhibitory effects of the isolates against selected foodborne pathogens, including *Escherichia coli*, *Staphylococcus aureus*, and *Bacillus cereus*, were evaluated using the agar well diffusion method. Mueller–Hinton agar plates (HiMedia Laboratories Pvt. Ltd., India) were swabbed with freshly prepared pathogenic cultures. Wells were aseptically prepared using sterile cork borers. Cell-free culture supernatants were obtained by centrifuging overnight cultures at 7000 rpm for 10 min, and approximately 0.1 mL of the supernatant was added to each well. The plates were then incubated at 37 °C for 24 h. The diameters of the inhibition zones surrounding the wells were measured after incubation (Chen et al., 2023).

### 2.7 Statistical analysis

All experiments were performed in triplicate, and the results are expressed as mean ± standard deviation (SD). Statistical analysis was performed using one-way analysis of variance (ANOVA) followed by Tukey’s honestly significant difference (HSD) test to determine significant differences among groups. Differences were considered statistically significant at *p* < 0.05. Data processing and graphical representation were performed using Microsoft Excel (Microsoft Corporation, United States), and statistical analysis was conducted using OriginPro software (OriginLab Corporation, United States).

## 3 Results

### 3.1 Physical and proximate characteristics of local and hybrid pearl millet

The physical properties of local and hybrid pearl millet are presented in Table 2, and the proximate composition of local and hybrid pearl millet is presented in Table 3. The physical properties of hybrid pearl millet show that it has slightly higher seed weight, bulk density, and hydration properties, such as hydration index, hydration capacity, swelling index, and swelling capacity, indicating comparatively higher hydration-related properties in hybrid pearl millet. Local pearl millet exhibited higher seed density and a higher germination rate (90%) compared with hybrid pearl millet (69%).

**Table 2:** Physical characteristics of local and hybrid pearl millets.

Characteristics	Local pearl millet	Hybrid pearl millet
Seed weight (g)	10.6 ± 0.12^a^	11.12 ± 0.04^b^
Seed density (g/mL)	0.83 ± 0.02^a^	0.74 ± 0.02^b^
Seed volume (g/mL)	0.035 ± 0.008^a^	0.031 ± 0.007^a^
Bulk density (g/mL)	0.890 ± 0.012^a^	0.910 ± 0.009^a^
Hydration index	0.005 ± 0.00^a^	0.007 ± 0.01^a^
Hydration capacity (g/seed)	0.007 ± 0.00^a^	0.009 ± 0.00^b^
Swelling index	6.89 ± 0.12^a^	8.64 ± 0.09^b^
Swelling capacity (mL/seed)	0.004 ± 0.00^a^	0.008 ± 0.00^b^
Germination percent (%)	90 ± 0.8^a^	69 ± 0.6^b^

Values are expressed as mean ± SD (*n* = 3). Different superscript letters within the same row indicate significant differences according to Tukey’s HSD test (*p* < 0.05).

**Table 3:** Proximate composition of local and hybrid pearl millet.

Proximate composition	Local pearl millet	Hybrid pearl millet
Moisture (%)	11.33 ± 0.043^a^	11.71 ± 0.09^b^
Total ash (%)	0.95 ± 0.06^a^	1.05 ± 0.02^a^
Crude fat (%)	3.82 ± 0.05^a^	3.96 ± 0.06^a^
Protein (%)	9.98 ± 0.26^a^	11.75 ± 0.30^b^
Crude fiber (%)	1.57 ± 0.03^a^	3.10 ± 0.04^b^

Values are expressed as mean ± SD (*n* = 3). Different superscript letters within the same row indicate significant differences according to Tukey’s HSD test (*p* < 0.05).

The local and hybrid millets had similar ash, moisture, and fat contents. However, hybrid millet had higher protein and fiber content than local millet. The comparatively lower crude fiber content in local pearl millet may influence the substrate characteristics during fermentation (Kumari et al., 2022). Fermented millet-based food materials are generally prepared using local millet, which has been reported to harbor local strains of lactic acid bacteria in fermented millet-based products (Tomar et al., 2025). Based on its comparatively lower crude fiber content, higher germination percentage, and traditional use in fermented millet-based foods, local pearl millet was selected as the substrate for the isolation of bacterial isolates for further functional characterization (Xu et al., 2026).

### 3.2 Isolation and screening of bacterial isolates

**Figure 1:**
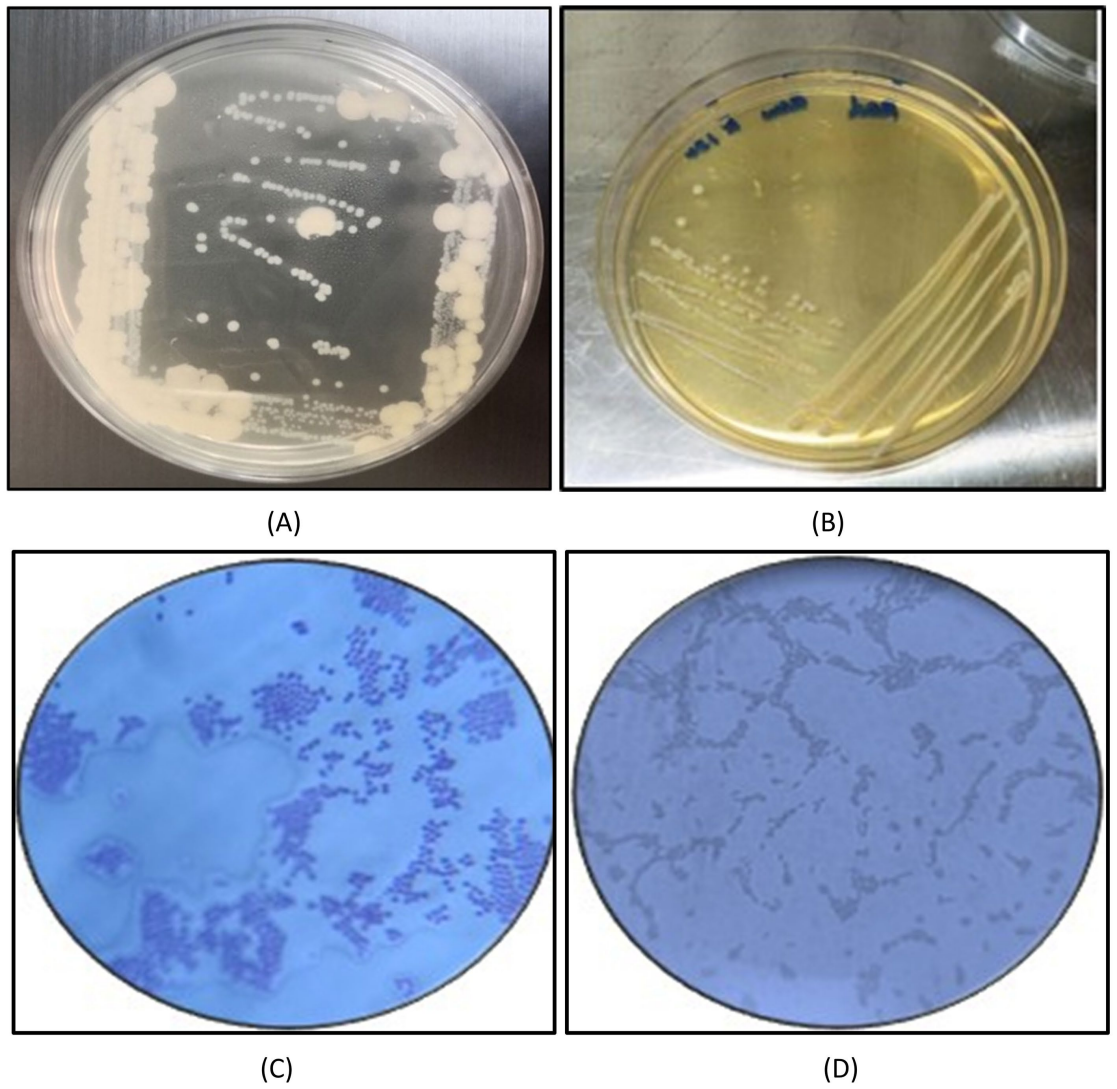
Isolation and purification of bacterial isolates from fermented pearl millet on MRS agar. **(A)** Mixed colonies during primary isolation; **(B)** pure culture obtained after repeated streaking; **(C)** Gram-positive cocci (*Pediococcus pentosaceus*); **(D)** Gram-positive rods (*Lactiplantibacillus plantarum*).

A total of 66 bacterial colonies with diverse morphologies were isolated from the fermented pearl millet samples (Figure 1). All isolates were initially screened based on Gram staining, catalase activity, and colony morphology. Among them, 33 isolates were Gram-positive, of which nine were catalase-negative. Based on these preliminary screening criteria, Gram-positive and catalase-negative isolates were selected for further characterization (Galli et al., 2022; Madushanka et al., 2025).

### 3.3 Biochemical characterization of selected isolates

The biochemical characteristics of the selected isolates are presented in Table 4. The isolates PJ2, PJ8, and PJ23 were positive for the methyl red (MR) test but negative for the Voges–Proskauer (VP) test. These isolates did not produce indole, H_2_S, or utilize citrate. Other isolates showed variable reactions, including H_2_S production, nitrate reduction, citrate utilization, or VP positivity. Based on their overall biochemical characteristics and functional potential, isolates PJ2, PJ8, and PJ23 were selected for further functional characterization. While these three isolates shared similar preliminary profiles during initial bench screening, their distinct taxonomic identities were subsequently resolved through molecular analysis.

**Table 4:** Biochemical characteristics of selected isolates.

Isolate	MR	VP	H_2_S	Indole	Gas	Ammonia	Nitrate	Citrate
PJ2	+	−	−	−	—	−	−	−
PJ4	+	−	+	−	+	−	−	−
PJ5	−	−	+	−	+	+	+	−
PJ6	−	−	+	−	−	−	+	+
PJ7	−	−	+	−	−	−	−	−
PJ8	+	−	−	−	—	−	−	−
PJ9	+	−	−	+	+	+	−	−
PJ11	−	+	+	−	−	−	+	−
PJ23	+	−	−	−	—	−	−	−

(+) Positive reaction; (−) Negative reaction.

### 3.4 Molecular identification

The selected isolates were subjected to 16S rRNA gene sequencing for molecular identification. The acquired nucleotide sequences were analyzed using the NCBI BLAST tool and compared with reference sequences available in the GenBank database.

**Figure 2:**
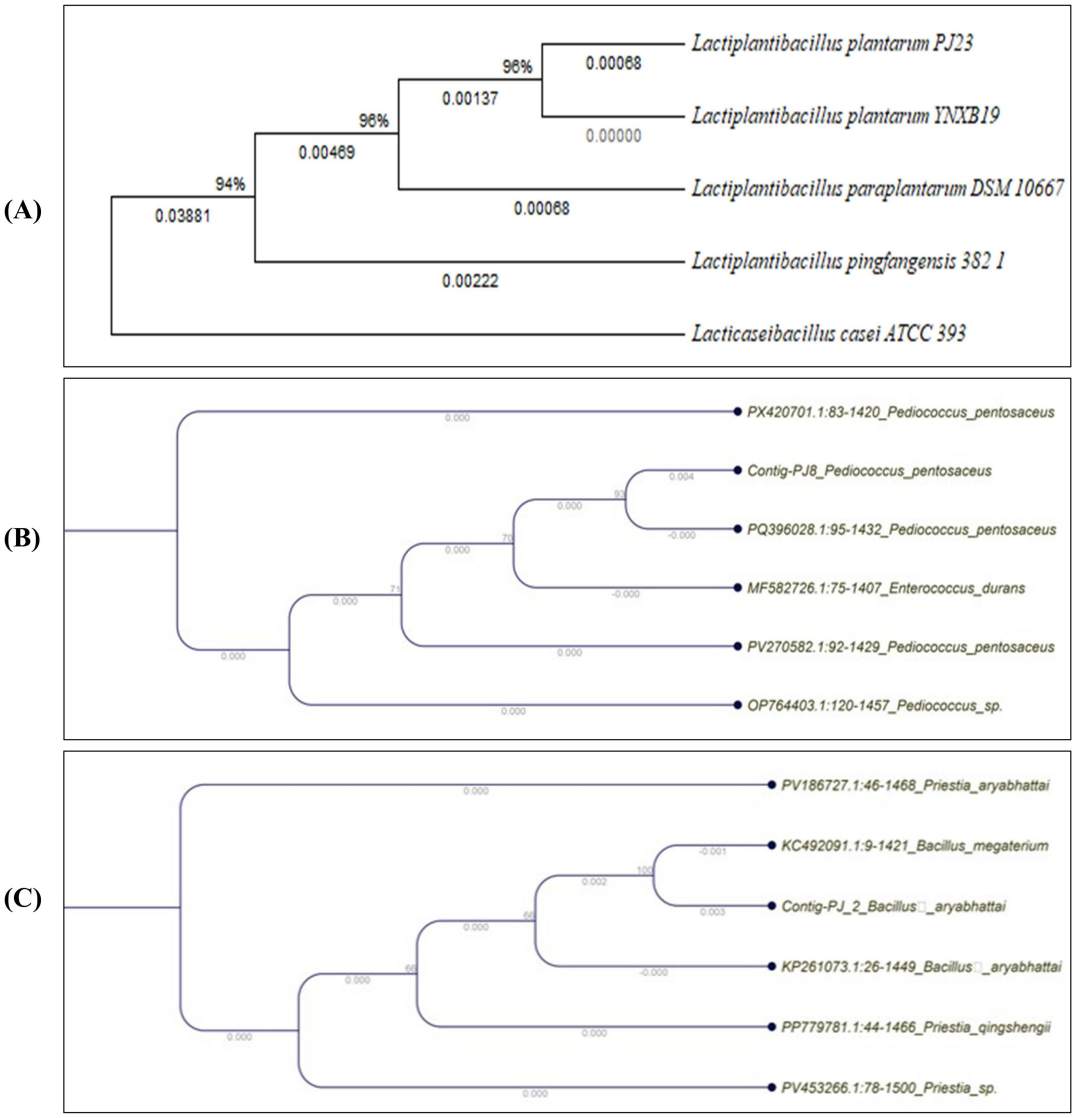
Phylogenetic analysis of selected isolates based on 16S rRNA gene sequences. **(A)** Phylogenetic tree of isolate PJ23 (*Lactiplantibacillus plantarum*); **(B)** phylogenetic tree of isolate PJ8 (*Pediococcus pentosaceus*); **(C)** phylogenetic tree of isolate PJ2 (*Bacillus aryabhattai*).

Based on sequence similarity analysis, isolate PJ2 was identified as *Bacillus aryabhattai* and deposited in the NCBI GenBank database under the accession number PX981827. Isolate PJ8 was identified as *Pediococcus pentosaceus* and deposited under the accession number PZ028018, whereas isolate PJ23 was identified as *Lactiplantibacillus plantarum* and deposited under the accession number PX983443. The detailed identification results, including sequence identity and GenBank reference IDs, are presented in Table 5. Although the preliminary screening strategy focused on isolates exhibiting phenotypic and biochemical characteristics commonly associated with lactic acid bacteria (LAB), isolate PJ2 exhibited LAB-associated characteristics during the screening process and was therefore selected for further characterization. However, subsequent 16S rRNA gene sequencing identified PJ2 as *Bacillus aryabhattai*, highlighting the limitations of relying solely on phenotypic and biochemical screening for LAB identification. Similar findings have been reported in studies involving fermented millet substrates, where isolates selected based on LAB-associated phenotypic traits were later identified as taxonomically diverse bacterial genera following molecular characterization (Woo et al., 2008; Divisekera et al., 2019). Phylogenetic analysis was performed to evaluate the evolutionary relationships between the isolates and closely related reference strains. The generated phylogenetic tree confirmed the taxonomic placement of the isolates within their respective families and supported the results of the sequence similarity analysis (Figures 2A–C).

**Table 5:** Identification of bacterial isolates from fermented pearl millet based on 16S rRNA gene sequence analysis using the NCBI BLAST algorithm.

Isolate	Identified species	Identity (%)	GenBank ID of reference strain	Accession number
PJ2	*Bacillus aryabhattai*	99.51	KP261073.1	PX981827
PJ8	*Pediococcus pentosaceus*	99.03	PQ396028.1	PZ028018
PJ23	*Lactiplantibacillus plantarum*	99.86	AB598940.1	PX983443

### 3.5 Acid tolerance

**Figure 3:**
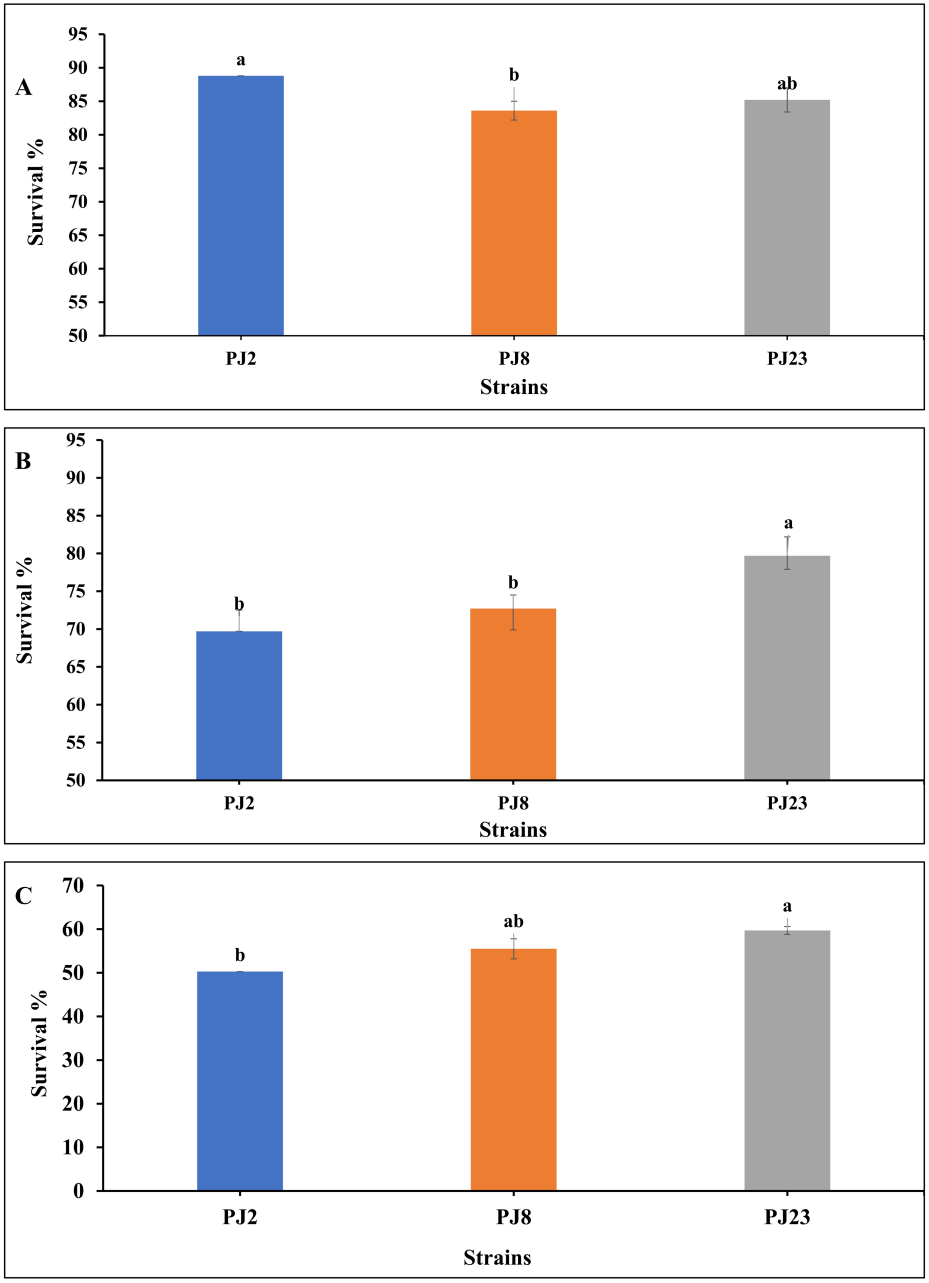
Survival percentage of selected isolates PJ2, PJ8, and PJ23 under acidic conditions at pH 2.0 after 1 h **(A)**, 2 h **(B)**, and 3 h **(C)** of exposure. Values represent mean ± SD (*n* = 3). Different superscript letters indicate significant differences (*p* < 0.05) according to one-way ANOVA followed by Tukey’s HSD test.

**Figure 4:**
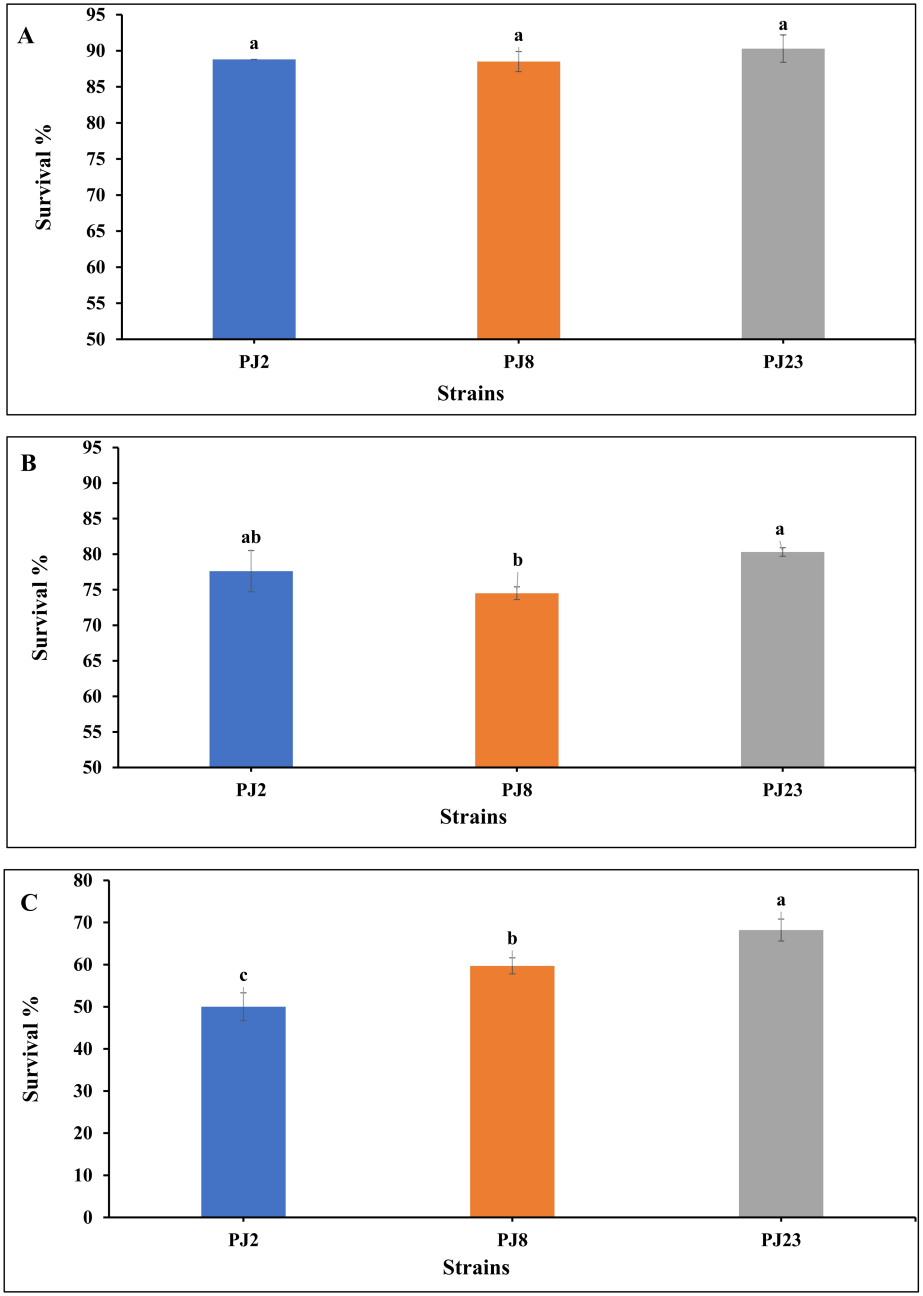
Survival percentage of selected isolates PJ2, PJ8, and PJ23 under acidic conditions at pH 4.0 after 1 h **(A)**, 2 h **(B)**, and 3 h **(C)** of exposure. Values represent mean ± SD (*n* = 3). Different superscript letters indicate significant differences (*p* < 0.05) according to one-way ANOVA followed by Tukey’s HSD test.

Survival under acidic pH conditions is commonly assessed during *in vitro* characterization of bacterial isolates. In this investigation, PJ2, PJ8, and PJ23 showed survival rates at acidic pH 2.0 and pH 4.0. Survival rates were from 83.6 to 88.8% when exposed to pH 2.0 for an hour. PJ2 and PJ8 were found to differ significantly, whereas PJ23 did not differ significantly from either isolate (*p* < 0.05). The survival pattern of the isolates under acidic conditions at pH 2.0 (Figure 3) and pH 4.0 (Figure 4), respectively. At 2 h, PJ23 showed significantly higher survival than PJ2 and PJ8, while no significant difference was observed between PJ2 and PJ8 (*p* > 0.05). These observations indicate survival under acidic conditions during the tested exposure period. However, following long-term exposure, survival rates were much lower, at 50.3–59.7% after 3 h. A reduction in survival was observed following prolonged exposure to acidic conditions (Surve et al., 2022). Similar survival patterns under prolonged acidic stress have been reported in several bacterial isolates associated with fermented foods (Gupta et al., 2021).

Acid tolerance also varies among strains. Lee et al. (2024) showed that *Lactiplantibacillus plantarum* LM1001 was viable under different pH conditions, but the type strain ATCC 14917 died under pH 2.5. This is similar to what was found among PJ2, PJ8, and PJ23 when they were exposed to acid stress under similar experimental conditions.

Among the isolates tested, PJ23 demonstrated a significantly higher survival rate than the other isolates examined at both pH levels, especially after 3 h of incubation (*p* < 0.05). Acid tolerance in *Lactiplantibacillus plantarum* may involve mechanisms related to intracellular pH regulation, stress response pathways, and maintenance of cellular homeostasis under acidic conditions (Wu et al., 2014). Acidic conditions can affect bacterial metabolic activity through intracellular acidification following the diffusion of protonated acids across the cell membrane, while acid resistance in *L. plantarum* has been associated with F_1_F_0_-ATPase activity and membrane-associated enzymes such as Cfa1, MleS, and HisD (Surve et al., 2022; Byun and Kim, 2023). In a similar context, *L. plantarum* strains MA2, B23, and GCC_19M1 have demonstrated high viability at mildly acidic pH (Tang et al., 2018; Nath et al., 2025).

On the other hand, *Pediococcus pentosaceus* PJ8 showed moderate survival under acidic conditions. Previous studies have reported acid and bile tolerance in several *P. pentosaceus* strains, including CRAG3 (Shukla and Goyal, 2014; Cao et al., 2016), as well as SC28, QK-1, RQ-1, and MQ-1 (Hu et al., 2021; Yang et al., 2020). However, the studies also revealed considerable variability among the strains. Such variability may be best explained by the genetic background and habitat of the strains (Xia et al., 2021). In the present study, PJ8 showed comparatively stable survival rates during the initial hour of exposure, followed by a gradual decrease over time. Higher survival rates were observed at pH 4.0 than at pH 2.0.

PJ2 (*Bacillus aryabhattai*) showed survival under acidic conditions, with 50.3 ± 2.3% survival after 3 h at pH 2.0. Earlier research showed that *Bacillus aryabhattai* HY1 had 16 ± 1% survival under similar conditions and duration (Pham et al., 2025). In the present study, PJ2 showed comparatively higher survival under the tested conditions. Although lactic acid bacteria are among the most widely studied bacterial groups in fermented foods, several *Bacillus* species have also been investigated for their functional characteristics because of their spore-forming ability, environmental resilience, and survival under gastrointestinal conditions (Vasiee et al., 2022; Pham et al., 2025)

After 3 h of exposure, significant differences in survival rates were observed among the isolates (*p* < 0.05). PJ23 exhibited the highest survival, while PJ8 showed intermediate survival and PJ2 showed the lowest survival under the tested conditions. The ability of the isolates to maintain more than 50% viability after 3 h at pH 2.0 suggests survival under simulated acidic conditions. Nonetheless, as stated by (Gupta et al., 2021), acid tolerance alone is insufficient to establish functional efficacy, and further safety and *in vivo* studies are required for comprehensive characterization.

### 3.6 Bile salt tolerance (0.3% bile)

**Figure 5:**
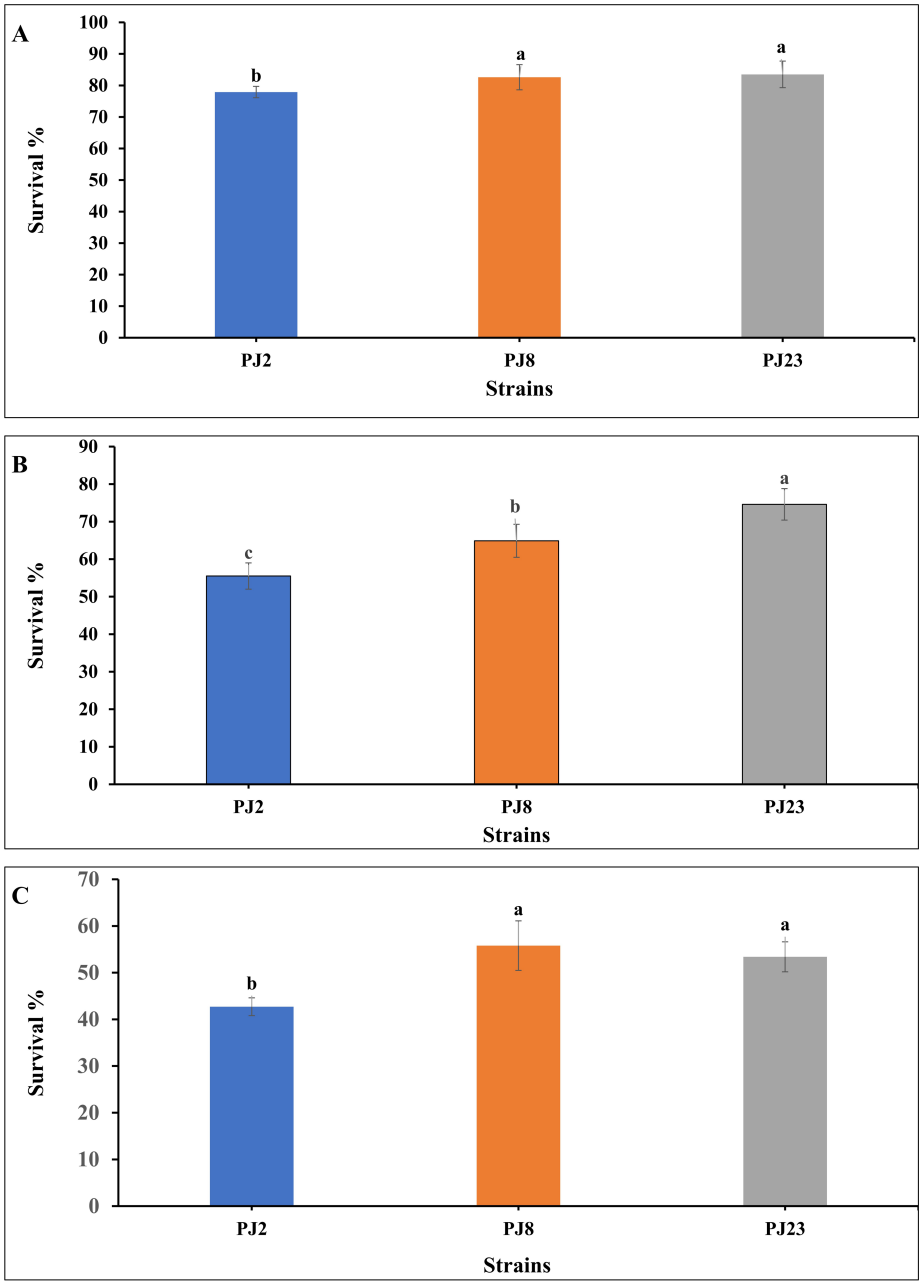
Survival rate (%) of selected isolates PJ2, PJ8, and PJ23 under 0.3% bile conditions after 1 h **(A)**, 2 h **(B)**, and 3 h **(C)** of exposure. Values are expressed as mean ± SD (*n* = 3). Different letters indicate significant differences (*p* < 0.05).

In the bile tolerance test, isolates PJ2 (*Bacillus aryabhattai*), PJ8 (*Pediococcus pentosaceus*), and PJ23 (*Lactiplantibacillus plantarum*) were exposed to 0.3% bile, and all of them survived the incubation period, indicating survival under bile stress conditions. The survival pattern of the isolates under bile stress is shown in Figure 5. After 1 h of incubation, PJ2, PJ8, and PJ23 had survival rates of 77.9 ± 1.8%, 82.6 ± 4.0%, and 83.5 ± 4.2%, respectively. PJ8 and PJ23 showed significantly higher survival than PJ2 at 1 h (*p* < 0.05). All isolates showed a gradual decline in survival percentage as exposure duration increased.

After 2 h of incubation, survival rates declined to 55.5 ± 3.5% for PJ2 (*B. aryabhattai*), 64.9 ± 4.4% for PJ8 (*P. pentosaceus*), and 74.6 ± 4.2% for PJ23 (*L. plantarum*). At 2 h, all isolates differed significantly, with PJ23 showing the highest survival, followed by PJ8 and PJ2 (*p* < 0.05). After 3 h, survival rates decreased to 42.7 ± 1.9%, 55.8 ± 5.3%, and 53.4 ± 3.2% for PJ2, PJ8, and PJ23, respectively. At 3 h, PJ8 and PJ23 showed significantly higher survival than PJ2, with no significant difference between them (*p* > 0.05). PJ23 showed higher survival during the initial incubation periods, while PJ8 exhibited slightly higher survival after prolonged exposure. Similar bile tolerance has been recorded in some *Lactiplantibacillus plantarum* strains (Hamon et al., 2011). For example, relative growth rates at 0.5% bile concentration ranged from 58 to 97% among different *L. plantarum* strains (Hamon et al., 2011), indicating variability in bile tolerance among strains. The survival pattern observed for PJ23 in the present study falls within this reported tolerance range. Likewise, similar tolerance to bile has been observed for strains of *Pediococcus pentosaceus*, including *P. pentosaceus* JBCC106 (Park et al., 2023).

Bile salts can affect bacterial survival by influencing cellular integrity and metabolic activity (Ruiz et al., 2013). The gradual decrease in viability observed over time indicates reduced survival with prolonged bile exposure. Nonetheless, all isolates maintained more than 40% viability after 3 h under the tested bile stress conditions (Hamon et al., 2011). Overall, PJ8 and PJ23 exhibited comparatively higher bile tolerance than PJ2 under the tested conditions. A rose jam-derived strain, *Pediococcus pentosaceus* MP13, also remained viable after 3 h of exposure to pH 2.0 and 0.5% (w/v) bile salts (Xia et al., 2021). Variability in survival under acidic and bile conditions has been reported among *P. pentosaceus* strains, which may be influenced by strain-specific characteristics and ecological adaptation (Xia et al., 2021).

*Lactiplantibacillus plantarum* has been reported to employ multiple mechanisms associated with bile stress resistance, including bile salt hydrolase activity, modification of membrane properties, protection against oxidative damage, and maintenance of proton motive force (Fidanza et al., 2021). These mechanisms may contribute to the maintenance of cellular integrity under bile stress conditions. In the present study, PJ23 showed comparatively higher survival during the early stages of bile exposure. A gradual decline in viability was observed with prolonged exposure to bile salts (Li et al., 2025). Overall, the isolates maintained baseline viability under the tested stress conditions, establishing their preliminary resilience *in vitro*.

### 3.7 Exopolysaccharide (EPS) production

When the strains were grown on MRS agar medium, the cultures of strains PJ8 and PJ23 showed the presence of ropiness along with smooth mucoidness and thread-like structures. Ropiness and mucoidness have commonly been associated with EPS-associated bacterial phenotypes in previous studies (Zaghloul and Ibrahim, 2022). Similar ropy phenotypes have been reported in *L. plantarum* strains isolated from fermented dairy products, such as LBIO1 and LBIO28 (Bachtarzi et al., 2020). It is important to note that LAB can produce structurally diverse EPS molecules differing in side chains, linkages, substitutions, and charges (Bachtarzi et al., 2020; Bibi et al., 2021). Exopolysaccharide production in lactic acid bacteria is strongly influenced by the genetic background of the strains, particularly the presence and expression of eps gene clusters (Zaghloul and Ibrahim, 2022). PJ23 showed mucoid and ropy colony characteristics under the tested *in vitro* conditions. However, EPS extraction and purification, quantitative estimation, molecular weight determination, monosaccharide composition analysis, and structural characterization using FTIR, NMR, SEM, and AFM analyses would be required to confirm exopolysaccharide production and related physicochemical characteristics (Yu et al., 2023).

### 3.8 Antioxidant activity (DPPH radical scavenging assay)

**Figure 6:**
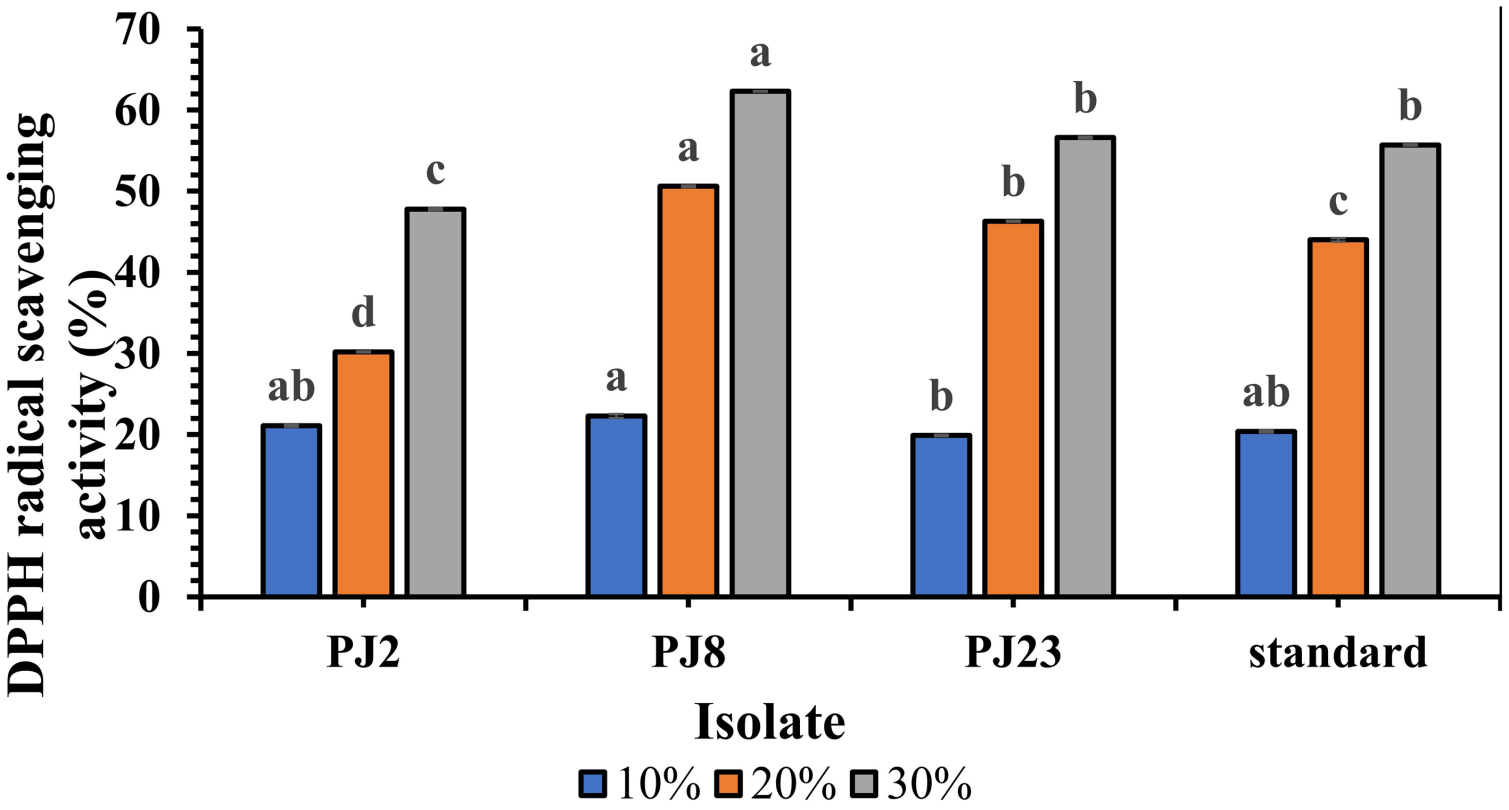
DPPH radical scavenging activity (%) of selected isolates PJ2, PJ8, and PJ23 compared with the standard at different concentrations (10, 20, and 30%). Values represent mean ± SD (*n* = 3). Different letters above the bars indicate significant differences among isolates within the same concentration (*p* < 0.05) according to Tukey’s HSD test.

The antioxidant activities of the isolates were investigated through a 2,2-diphenyl-1-picrylhydrazyl (DPPH) free radical scavenging assay. This assay determines the antioxidant potential of a compound or a mixture of compounds in scavenging DPPH free radicals. As shown in Figure 6, all the isolates exhibited DPPH free radical scavenging activity, indicating the presence of antioxidant activity in the culture filtrates of the isolates. The proportion of inhibition also increased with concentration. That is, radical scavenging activity was low at 10%, increased at 20%, and peaked at 30%. This suggests that higher concentrations of culture filtrate may contain greater levels of antioxidant compounds capable of scavenging DPPH free radicals (Baliyan et al., 2022). Among the tested strains, PJ8, a *Pediococcus pentosaceus* strain, showed significantly higher antioxidant activity with a maximum inhibition value of around 62.3% at 30% concentration, which is higher than that of PJ2 and PJ23. On the other hand, PJ2, a *Bacillus aryabhattai* strain, showed the lowest scavenging activity among all concentrations, indicating comparatively lower DPPH scavenging activity. This difference was statistically significant compared to other isolates within the same concentration (*p* < 0.05). PJ23, a *Lactiplantibacillus plantarum* strain, showed intermediate antioxidant activity.

Bacterial isolates in culture media can create a variety of metabolites, including peptides, exopolysaccharides, organic acids, and phenolic compounds, which may contribute to radical scavenging activity (Yamamoto et al., 2019). These metabolites can contribute electrons or hydrogen atoms, stabilize free radicals, and reduce oxidative processes. Han et al. (2017) reported that intact cells of certain lactic acid bacterial strains exhibited higher antioxidant activity than cell-free extracts and supernatants, indicating that cell surface-associated components may contribute to antioxidant activity in a strain-dependent manner. In addition to cell-associated factors, extracellular metabolites present in culture filtrates may also contribute to DPPH radical scavenging activity under *in vitro* conditions (Kim et al., 2019).

Comparative studies of different lactic acid bacterial strains have shown that *Pediococcus pentosaceus* AR243, which was isolated from Chinese traditionally fermented foods, showed potent hydroxyl and DPPH radical scavenging ability, contributing to lipid peroxidation inhibition (Lin et al., 2018). Łepecka et al. (2023) reported strain-dependent antioxidant activity among lactic acid bacteria isolated from fermented meat products, with *Pediococcus pentosaceus* KL14 showing the highest DPPH radical scavenging activity (36.05%), whereas *Lactiplantibacillus plantarum* SCH1 exhibited comparatively lower activity (21.88%). These findings are generally consistent with the present study, where *Pediococcus pentosaceus* PJ8 exhibited higher antioxidant activity than *Lactiplantibacillus plantarum* PJ23, indicating strain-dependent variation in antioxidant-associated characteristics among bacterial isolates. Antioxidant characteristics similar to those seen in lactic acid bacteria have been reported, with DPPH radical scavenging activity ranging between 34 and 83% depending on the strain (Lin et al., 2018). In the present study, antioxidant activity ranged from 19.9 to 62.3%, with significant differences observed among isolates at each concentration level (*p* < 0.05). Overall, the results indicate measurable DPPH radical scavenging activity under the tested *in vitro* conditions.

### 3.9 Cell surface hydrophobicity

**Figure 7:**
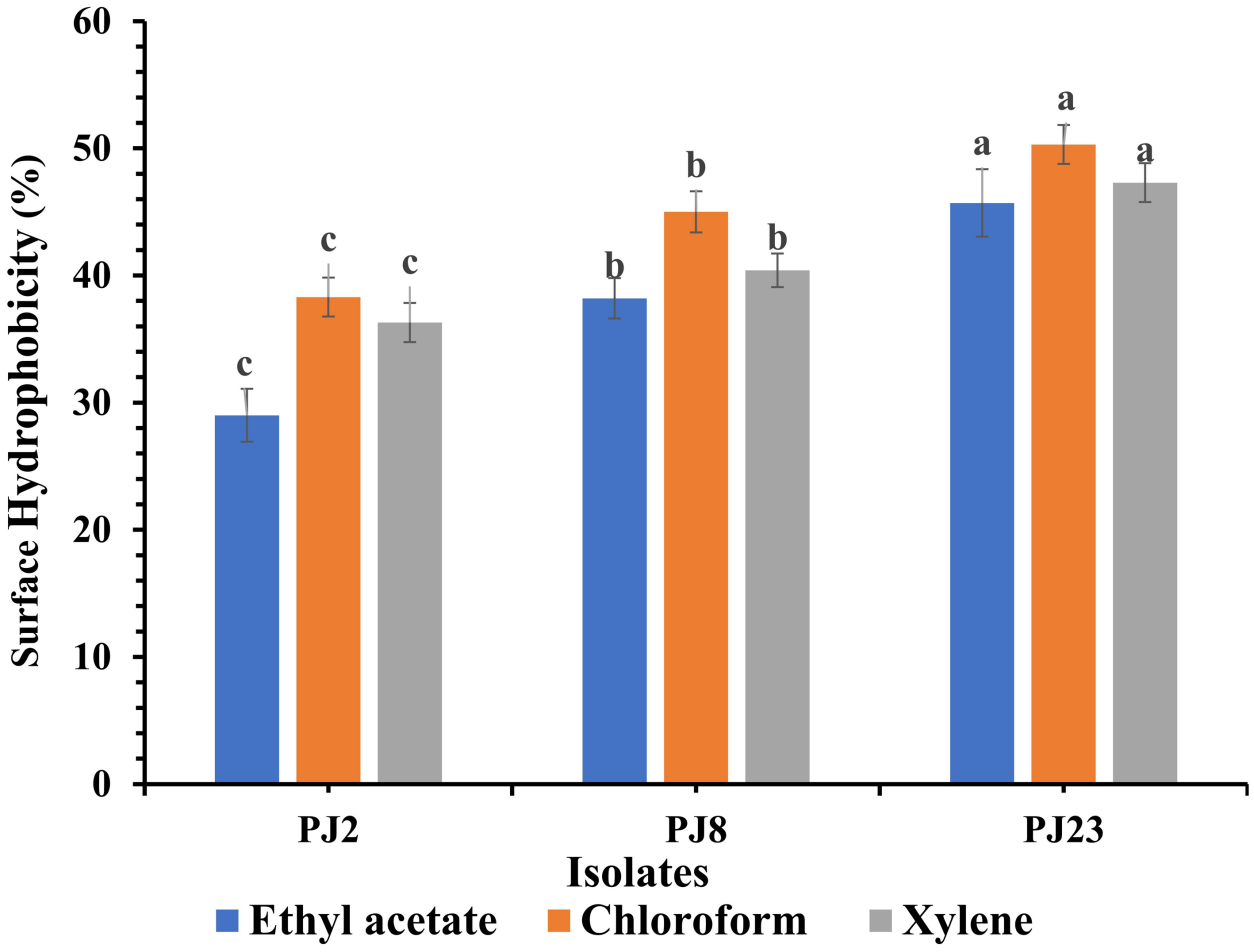
Cell surface hydrophobicity (%) of selected isolates PJ2 (*Bacillus aryabhattai*), PJ8 (*Pediococcus pentosaceus*), and PJ23 (*Lactiplantibacillus plantarum*) using ethyl acetate, chloroform, and xylene. Values represent mean ± SD (*n* = 3). Different superscript letters indicate significant differences (*p* < 0.05).

Ethyl acetate, xylene, and chloroform were used to evaluate the preliminary surface hydrophobicity and acid–base interaction characteristics of the isolates (Shaffique et al., 2022). These surface interaction characteristics are associated with the physicochemical composition of the microbial cell surface and Lewis acid–base interactions (Dias et al., 2013). The isolates exhibited varying levels of cell surface hydrophobicity under the tested in vitro conditions (Figure 7). Among the isolates, PJ23 showed the highest hydrophobicity with ethyl acetate (45.7 ± 2.65%), chloroform (50.3 ± 1.53%), and xylene (47.3 ± 1.53%). PJ23 showed significantly higher hydrophobicity values than PJ8 and PJ2 across all solvents, while PJ8 also exhibited significantly higher hydrophobicity values than PJ2 (*p* < 0.05). The moderate hydrophobicity values observed in the present study are comparable to those reported by (Kaushik et al., 2009), who reported hydrophobicity values of 47% in *Lactobacillus johnsonii* LA1, 57–58% in *L. acidophilus* LA7, and 37.1–37.7% in *L. plantarum*.

The hydrophobicity of isolate PJ8 was relatively high, with ethyl acetate (38.2 ± 1.59%), chloroform (45.0 ± 1.62%), and xylene (40.4 ± 1.32%). However, isolate PJ2 (*B. aryabhattai*) displayed lower values, with ethyl acetate (29.0 ± 2.08%), chloroform (38.3 ± 1.53%), and xylene (36.3 ± 1.53%). In contrast, (Schillinger et al., 2005) reported considerably higher hydrophobicity values ranging between 74 and 95% in *L. acidophilus*, while (Liu et al., 2021) reported hydrophobicity values ranging from 20.95 to 90.09% in *Lactobacillus plantarum* strains and from 41.87 to 98.03% in *Lactobacillus brevis* strains, further highlighting the variability in hydrophobicity reported among different bacterial strains. Cell surface hydrophobicity is considered an important physicochemical property that may influence microbial surface interaction characteristics (Krasowska and Sigler, 2014). However, microbial adhesion in the gastrointestinal environment is a complex, multifactorial phenomenon (Racioppo et al., 2025). These in vitro solvent-partitioning assays serve as a preliminary screening tool and do not directly confirm intestinal adhesion, mucosal colonization ability, or technological performance in food systems. Nevertheless, the assay provides useful preliminary information regarding the surface interaction characteristics of the isolates under in vitro conditions.

### 3.10 Antibiotic resistance profiling

The antibiotic susceptibility of the isolated strains PJ2 (*Bacillus aryabhattai*), PJ8 (*Pediococcus pentosaceus*), and PJ23 (*Lactiplantibacillus plantarum*) was tested by the disc diffusion method for the five commonly used antibiotics, such as ampicillin (AMP), erythromycin (E), gentamicin (G), streptomycin (S), and kanamycin (K) (Table 6). The zone of inhibition for each strain was determined by the millimeter scale and expressed as the mean ± SD. Antibiotic susceptibility was interpreted according to antibiotic-specific inhibition zone diameter criteria described by (Charteris et al., 1998). PJ23 (*Lactiplantibacillus plantarum*) showed the largest inhibition zone against ampicillin (50 ± 5 mm), followed by kanamycin (31.7 ± 7.6 mm). The isolate also showed inhibition zones against erythromycin (21.7 ± 10.4 mm), gentamicin (18.3 ± 7.6 mm), and streptomycin (15 ± 5 mm). Earlier studies (Katiku et al., 2022) reported similar antibiotic susceptibility patterns in *Lactiplantibacillus plantarum* strains, with inhibition zones ranging from 13.5 ± 0.71 mm for ampicillin to 17.0 ± 1.41 mm for gentamicin. The PJ8 *Pediococcus pentosaceus* showed larger inhibition zones against ampicillin. This was followed by inhibition zones against gentamicin (21.7 ± 2.9 mm) and streptomycin (17.7 ± 6.8 mm). The isolate exhibited moderate susceptibility to erythromycin and kanamycin, as indicated by comparatively smaller inhibition zones. Past studies have reported similar antibiotic susceptibility patterns. In these studies, the size of the inhibition zone formed by ampicillin varied from 11 mm to 39 mm, while that formed by gentamicin varied from 15 mm to 31 mm (Singla et al., 2018). The inhibition zone values observed for PJ8 *Pediococcus pentosaceus* are in line with the antibiotic susceptibility patterns shown by *Pediococcus* species.

**Table 6:** Antibiotic susceptibility profile of bacterial isolates determined by the disc diffusion method.

Antibiotic	PJ2 (*Bacillus aryabhattai*)	PJ8 (*Pediococcus pentosaceus*)	PJ23 (Lactiplantibacillus plantarum)
Ampicillin (AMP)	R (no inhibition zone)	S (40 ± 5)	S (50 ± 5)
Erythromycin (E)	R (11.7 ± 7.6)	MS (17 ± 5)	S (21.7 ± 10.4)
Gentamicin (G)	S (25 ± 5)	S (21.7 ± 2.9)	S (18.3 ± 7.6)
Streptomycin (S)	MS (11.7 ± 7.6)	S (17.7 ± 6.8)	S (15 ± 5)
Kanamycin (K)	MS (14.3 ± 4.0)	MS (15 ± 5)	S (31.7 ± 7.6)

Values represent inhibition zone diameter (mm) expressed as mean ± SD (*n* = 3). Antibiotic susceptibility was interpreted according to antibiotic-specific interpretative zone diameter standards described by (Charteris et al., 1998): AMP: R ≤ 12, MS 13–15, S ≥ 16; E: R ≤ 13, MS 14–17, S ≥ 18; G: R ≤ 12, S ≥ 13; S: R ≤ 11, MS 12–14, S ≥ 15; K: R ≤ 13, MS 14–17, S ≥ 18.

On the other hand, *PJ2 Bacillus aryabhattai* showed no inhibition zone for ampicillin, indicating resistance. Resistance to erythromycin was also observed, with an inhibition zone of 11.7 ± 7.6 mm. The isolate showed susceptibility to gentamicin (25 ± 5 mm) and moderate susceptibility to kanamycin (14.3 ± 4.0 mm). Streptomycin exhibited an inhibition zone of 11.7 ± 7.6 mm, indicating comparatively lower susceptibility toward this antibiotic. However, further MIC-based analysis and genomic characterization would be required for a comprehensive assessment of antibiotic resistance determinants in the isolates.

### 3.11 Antagonistic activity against foodborne pathogens

**Figure 8:**
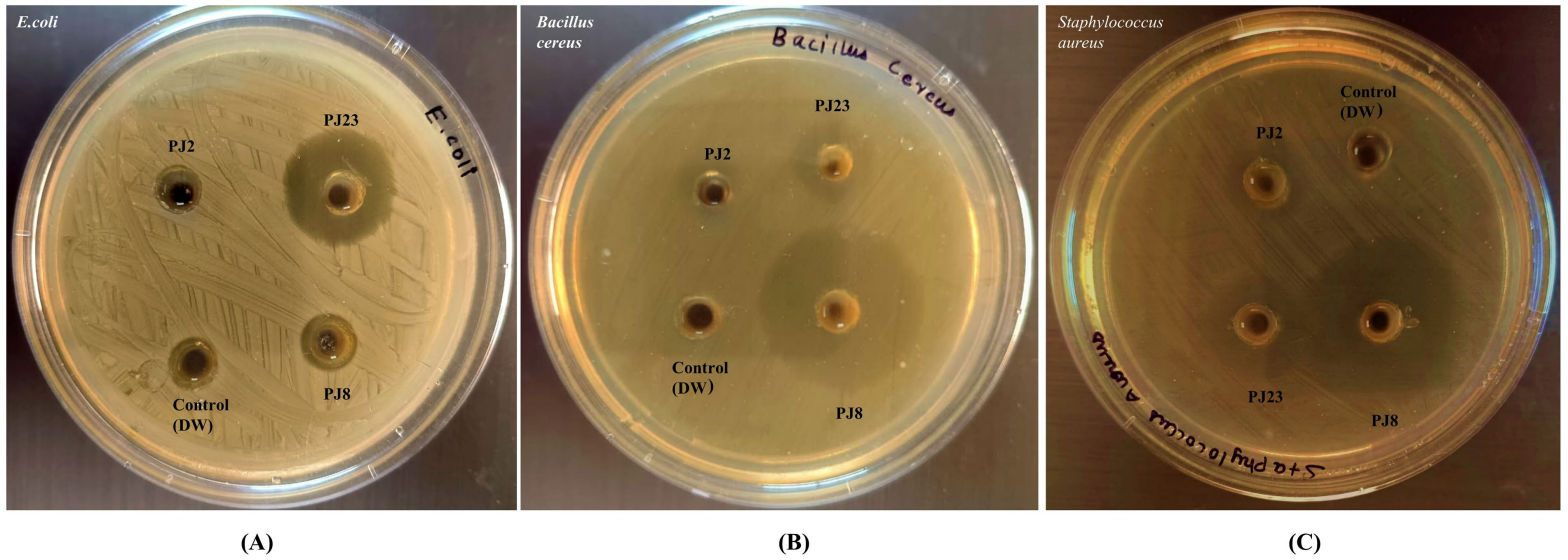
Antagonistic activity of selected isolates against foodborne pathogens using the agar well diffusion method. **(A)** *Escherichia coli*, **(B)** *Bacillus cereus*, and **(C)** *Staphylococcus aureus*.

The antagonistic activity of the isolates PJ2 *Bacillus aryabhattai*, PJ8 *Pediococcus pentosaceus*, and PJ23 *Lactiplantibacillus plantarum* was tested against the foodborne pathogens *Escherichia coli*, *Bacillus cereus*, and *Staphylococcus aureus* using the agar well diffusion method (Figure 8). Among the tested strains, PJ23 (*Lactiplantibacillus plantarum*) had the largest inhibitory effect against *Escherichia coli*, with the largest inhibition zone measuring 29.3 ± 4.0 mm (Table 7). PJ8 (*Pediococcus pentosaceus*) showed larger inhibition zones against the Gram-positive bacteria *Bacillus cereus* and *Staphylococcus aureus*, resulting in inhibition zones of 35 ± 5 mm and 40 ± 5 mm, respectively (Table 7). PJ23 also showed inhibitory effects against the Gram-positive bacteria, but the inhibition zones were smaller, measuring 16.7 ± 2.9 mm against *Bacillus cereus* and 21.7 ± 7.6 mm against *Staphylococcus aureus.*

**Table 7:** Antagonistic activity of bacterial isolates against foodborne pathogens determined by the agar well diffusion method.

Isolate	*Escherichia coli*	*Bacillus cereus*	*Staphylococcus aureus*
PJ2 (*Bacillus aryabhattai*)	10 ± 5^a^	8.3 ± 2.9^a^	8.3 ± 2.9^a^
PJ8 (*Pediococcus pentosaceus*)	16.7 ± 2.9^ab^	35 ± 5^c^	40 ± 5^c^
PJ23 (*Lactiplantibacillus plantarum*)	29.3 ± 4.0^b^	16.7 ± 2.9^b^	21.7 ± 7.6^b^

Values represent inhibition zone diameter (mm) expressed as mean ± SD (*n* = 3). Different superscript letters within the same column indicate significant differences according to Tukey’s HSD test (*p* < 0.05).

The inhibition zones varied significantly among the isolates depending on the pathogen tested. The observed differences in inhibition levels between Gram-positive and Gram-negative pathogens could be related to structural differences in their cell envelopes, which may influence susceptibility under the tested *in vitro* conditions (Silhavy et al., 2010; Belay et al., 2024). PJ2 (*Bacillus aryabhattai*) demonstrated lower antagonistic activity against all tested pathogens. PJ23 showed significantly higher inhibition against *Escherichia coli*, whereas PJ8 exhibited significantly higher inhibition against Gram-positive pathogens (*p* < 0.05).

Also, *Pediococcus* isolated from cheese has shown inhibition against *Bacillus cereus* ATCC 1178 (Gurira and Buys, 2005). This is in agreement with the higher inhibitory activity observed in the current study for PJ8, *Pediococcus pentosaceus*, against the Gram-positive pathogens. Comparable inhibitory activity (Gouthami et al., 2024) has been reported in LAB isolated from fermented millet and other cereal-based substrates, where cell-free supernatants exhibited inhibition zones ranging from 7.1 to 15.1 mm against *Escherichia coli* and approximately 10 mm against *Staphylococcus aureus*. Similarly, millet-derived LAB isolates reported by (Mohanan et al., 2025) demonstrated antagonistic activity against *Escherichia coli* and *Salmonella typhimurium*, with inhibition levels ranging from 77.5 ± 3.0% to 98.8 ± 0.5% depending on the isolate and concentration of the cell-free supernatant.

Antagonistic activity of LAB cell-free supernatants against enteric pathogens has also been reported by (Divyashree et al., 2024) in millet-associated LAB isolates, with inhibition ranging from 51.8 to 95.2% against *Escherichia coli* and 44.0 to 67.1% against *Staphylococcus aureus,* further supporting the antagonistic activity observed in millet-derived LAB isolates. The study on *L. plantarum* LY-21 revealed that the cell-free supernatant (CFS) exhibited antagonistic activity against ten Gram-positive and Gram-negative pathogens (Du et al., 2025). Overall, these findings provide preliminary evidence of the antagonistic activity of the isolates against foodborne pathogens (Kiani et al., 2021; Fijan, 2023). However, further studies involving pH-neutralized cell-free supernatants and characterization of antimicrobial compounds are necessary to clarify the specific compounds and mechanisms underlying the observed inhibitory activity (D’Carmo Sodré et al., 2026).

## 4 Conclusion

Fermented pearl millet was investigated as a source of bacterial isolates. From the fermented substrate, 66 colonies were initially isolated, and subsequent biochemical and molecular characterization identified three isolates: *Bacillus aryabhattai* (PJ2), *Pediococcus pentosaceus* (PJ8), and *Lactiplantibacillus plantarum* (PJ23). The isolates showed survival under acidic pH and bile salt stress conditions and exhibited measurable antioxidant activity, antagonistic activity against selected foodborne pathogens, EPS-associated phenotypic characteristics, and cell surface hydrophobicity under the tested in vitro conditions. Among the tested isolates, *Lactiplantibacillus plantarum* PJ23 exhibited comparatively higher values in several evaluated in vitro characteristics under the tested conditions, while *Pediococcus pentosaceus* PJ8 showed comparatively higher antioxidant activity. Overall, the findings demonstrate that fermented pearl millet harbors bacterial isolates exhibiting preliminary in vitro characteristics under the tested conditions. However, these observations are based only on preliminary in vitro evaluation, and further studies involving comprehensive taxonomic analysis, safety assessment, metabolite characterization, stability studies, and *in vivo* evaluation are required for further characterization of the isolates.

## Data Availability

The datasets presented in this study can be found in online repositories. The names of the repository/repositories and accession number(s) can be found in the article/supplementary material.
